# Association between SIRT1 Gene Polymorphisms and Breast Cancer in Egyptians

**DOI:** 10.1371/journal.pone.0151901

**Published:** 2016-03-21

**Authors:** Sherine M. Rizk, Nancy N. Shahin, Olfat G. Shaker

**Affiliations:** 1 Department of Biochemistry, Faculty of Pharmacy, Cairo University, Cairo, Egypt; 2 Department of Medical Biochemistry and Molecular Biology, Faculty of Medicine, Cairo University, Cairo, Egypt; China Medical University, TAIWAN

## Abstract

**Background:**

Breast cancer is reported to cause the highest mortality among female cancer patients. Previous studies have explored the association of silent mating-type information regulator 2 homolog 1 (SIRT1) gene expression with prognosis in breast cancer. However, no studies exist, so far, on the role of SIRT1 gene polymorphism in breast cancer risk or prognosis. The present study aimed to assess the association between SIRT1 gene polymorphisms and breast cancer in Egyptians.

**Methods:**

The study comprised 980 Egyptian females divided into a breast cancer group (541 patients) and a healthy control group (439 subjects). SIRT1 gene single nucleotide polymorphisms (SNPs) rs3758391, rs3740051 and rs12778366 were genotyped using real-time polymerase chain reaction (RT-PCR). Allelic and genotypic frequencies were determined in both groups and association with breast cancer and clinicopathological characteristics was assessed.

**Results:**

Breast cancer patients exhibited elevated serum SIRT1 levels which varied among different tumor grades. SIRT1 rs3758391 and rs12778366 TT genotypes were more frequent, exhibited higher SIRT1 levels than CC and CT genotypes and were associated with histologic grade and lymph node status. SIRT1 rs12778366 TT genotype also correlated with negative estrogen receptor (ER) and progesterone receptor (PR) statuses. The T allele frequency for both SNPs was higher in breast cancer patients than in normal subjects. Combined GG and AG genotypes of rs3740051 were more frequent, showed higher serum SIRT1 levels than the AA genotype, and were associated with ER and PR expression. Furthermore, inheritance of the G allele was associated with breast cancer.

**Conclusions:**

Our findings reveal that rs3758391 and rs12778366 polymorphisms of SIRT1 gene are associated with breast cancer risk and prognosis in the Egyptian population.

## Introduction

Sirtuin 1 (SIRT1) is 1 of 7 members of the sirtuin family of nicotinamide adenine dinucleotide (NAD^+^)-dependent class III histone deacetylase that are human homologues of yeast silent mating-type information regulator 2 (sir2) [1]. It mediates the deacetylation of various substrates including p53, forkhead box class O (FOXO), peroxisome proliferator activated receptors co-activator 1α (PGC1α) and other proteins, and thus regulates diverse physiological processes including aging, genomic stability and metabolism [2]. Misregulation of SIRT1 is implicated biochemically and genetically in diabetes and has been proposed as a therapeutic target in neurodegeneration, osteoarthritis and cardiovascular disease [3–7]. Therefore, SIRT1 is a multifunctional protein that plays a central role in various pathways. However, the role of SIRT1 in cancer has not been clearly defined.

Up-regulation of SIRT1 has been reported in various human malignancies including breast cancer [8], prostate cancer [9], acute myeloid leukemia [10], and primary colon cancer [11]. Based on the elevated levels of SIRT1 in cancers, it was hypothesized that SIRT1 serves as a tumor promoter [12]. In contrast, Wang et al. [13] analyzed a public database and found that SIRT1 expression was reduced in many other types of cancers, including glioblastoma, bladder carcinoma, prostate carcinoma and ovarian cancers as compared to the corresponding normal tissues. Their further analysis of 44 breast cancer and 263 hepatic carcinoma cases also revealed reduced expression of SIRT1 in these tumors [13]. These data suggest that SIRT1 acts as a tumor suppressor rather than a promoter in these tissues. The apparent opposed roles of SIRT1 seem contradictory but the multiple functions of SIRT1 made this possible. SIRT1 can negatively regulate multiple pathways including both tumor suppressors (p53, FOXO) and oncogenic proteins (survivin, β-catenin, NF-κB). The role of SIRT1 in tumorigenesis may well depend on the temporal and spacial distribution of different SIRT1 upstream regulators and downstream targets [14]. Roth and Chen [15] proposed that SIRT1 may act as a genome caretaker in normal cells and therefore suppress tumorigenesis. However, upon oncogenic events, tumor cells co-opt SIRT1-regulated cellular pathways to promote unabated proliferation, progression, resisting death signals, and genetic/epigenetic evolution. Overall, the role of SIRT1 as either a tumor promoter or a tumor suppressor is still under investigation, and more intense research is needed in order to understand the complex role of SIRT1 in tumorigenesis.

Breast cancer is reported to cause the highest mortality among female cancer patients. Over a million women worldwide are diagnosed with breast cancer every year, and another 400,000 are reported to succumb to the disease [16]. The pathogenesis of breast cancer is multifactorial; however, the association of SIRT1 expression with the clinical characteristics and prognosis in breast cancer has not been fully identified.

Epigenetic alterations including histone modifications are critical for breast carcinogenesis [17]. Indeed, promoter region variants may account for differential SIRT1 expression, rendering individuals susceptible to certain pathologies [18–21]. Summarizing all these data, one may assume that human SIRT1 expression and SIRT1 genetic variants may play a role in breast cancer clinicopathological features together with disease progression. So far, no studies exist on the involvement of SIRT1 gene polymorphism in breast cancer. We, therefore, conducted the present study to investigate SIRT1 genetic variants in a series of breast cancer cases as well as controls to determine whether SIRT1 polymorphism influences the risk for the development of breast cancer and prognosis.

## Materials and Methods

### Subjects

The present study was conducted on 541 blood samples provided by breast cancer patients, who were clinically diagnosed with breast cancer and confirmed by mammography and surgical biopsies at General Surgery Department at the Faculty of Medicine, Cairo University during the period from June 2013 to May 2014. The patient age range was 37–69 years, with a mean age of 52.3 years. No patients received antihormonal treatment, chemotherapy or radiotherapy prior to participation in the study. In addition, 439 age-matched healthy female volunteers (normal control group) were enrolled. They were recruited from healthy subjects admitted to the same hospital. They did not suffer hypertension, liver or renal diseases. They had no palpable breast masses and received no contraceptives. All clinicopathological data were collected from medical records for the analysis of differences among genotypes (Table 1). Written informed consent, approved by the Ethics Committee of Faculty of Pharmacy, Cairo University, was obtained from all subjects enrolled in the study.

**Table 1:** Demographic, laboratory and clinical data in control and breast cancer groups. Data are expressed as mean ± S.E., or number and percentage, n (%). ALT, alanine transaminase; AST, aspartate transaminase, ALP, alkaline phosphatase; T. Bil, total bilirubin; † Tumors were graded according to the modified Bloom–Richardson system; T size of the tumor, T1 (< 2 cm), T2 (≥ 2–5 cm), T3 (≥ 5 cm), T4 (infiltration of the chest wall/skin), N regional lymph node involvement, N1 cancer has spread to 1 to 3 axillary lymph node(s), and/or tiny amounts of cancer are found in internal mammary lymph nodes on lymph node biopsy, N2 cancer has spread to 4 to 9 axillary lymph nodes, or cancer has enlarged the internal mammary lymph nodes, N3 cancer has spread to axillary lymph nodes, the internal mammary lymph nodes and/or infraclavicular and supraclavicular lymph nodes; ER, estrogen receptor; PR, progesterone receptor.

Variables	Control group	Breast cancer group	p value
**Number**	439	541	
**Age (years)**	49.86 ± 1.09	52.33 ± 0.87	0.074
**ALT (IU/L)**	25.60 ± 0.41	28.30 ± 2.03	0.073
**AST (IU/L)**	23.40 ± 0.82	25.10 ± 1.68	0.310
**ALP (IU/L)**	88.02 ± 2.70	87.00 ± 9.59	0.892
**T. Bil (mg/dl)**	0.59 ± 0.02	0.61 ± 0.04	0.785
**Tumor type**			
**Duct**	-	469 (86.7)	-
**Others**	-	72 (13.3)	-
**Histological grade †**			
**1**	-	35 (6.5)	-
**2**	-	409 (75.5)	-
**3**	-	97 (18)	-
**Tumor-node-metastasis (TNM) classification**			
***Tumor size***			
**T2**	-	347 (64.1)	-
**T3**	-	187 (35.6)	-
**T4**	-	7 (1.3)	-
***Lymph node invasion***			
**N1**	-	108 (20)	-
**N2**	-	295 (54.5)	-
**N3**	-	138 (25.5)	-
***Metastasis***			
**Absent**	-	422 (78)	-
**Present**	-	119 (22)	-
**ER status**			
**Negative**	-	444 (82.1)	-
**Positive**	-	97 (17.9)	-
**PR status**			
**Negative**	-	452 (83.5)	-
**Positive**	-	89 (16.5)	-

Peripheral blood samples were divided into two tubes. One tube was used for separation of serum for the estimation of SIRT1 and routine laboratory tests. The other tube contained EDTA and was used for DNA extraction and SIRT1 genotyping analysis. All samples were handled at 4°C until they were stored for final analysis at -80°C. DNA extractions were performed simultaneously for all samples.

### Biochemical Investigations

Blood samples collected from all volunteers were immediately centrifuged at 4°C; the serum supernatants were frozen at -20°C. Alanine transaminase (ALT), aspartate transaminase (AST), alkaline phosphatase (ALP) and total bilirubin (T. Bil) assays were performed using QCA kits (Quimica Clinica Aplicada S.A. kits. Aspartado, Amposta, Spain) according to the methods of Reitman and Frankel [22], Babson et al. [23] and Jendrassik and Grof [24], respectively. Tumor estrogen receptor (ER) and progesterone receptor (PR) had been determined immunohistochemically [25]. Serum SIRT1 level was estimated using an enzyme-linked immunosorbent assay (ELISA) kit provided by Shanghai SunRed Biological Technology (Shanghai, China).

### DNA Extraction and Genotyping of SIRT1 Gene Single Nucleotide Polymorphisms (SNPs)

#### DNA Extraction

DNA was extracted from whole blood of patient and control groups using QIA-amplification extraction kit (Qiagen, Venlo, Limburg, Netherlands), following the manufacturer’s instructions. DNA samples were quantitated using the NanoDrop®-1000 spectrophotometer (NanoDrop Technologies, Inc., Wilmington, USA).

#### Genotyping

Genotyping was determined using real time PCR. Single nucleotide polymorphisms (SNPs) of rs3758391, rs3740051 and rs12778366 (Cat No. 4351379) were analyzed in the extracted DNA by using specific primers and Taqman probes (Taqman genotyping assays, Applied Biosystems, Foster City, CA). We used Tagger software (http://www.broadinstitute.org/mpg/tagger/) to select tag SNPs in the SIRT1 region plus 0.5 Kb downstream, and 30 Kb upstream (NCBI Build 35/UCSC hg17), the minor allele frequency > 5% in the Caucasian population and r^2^ > 1 as criteria. The 3 studied SNPs were located in the 5’ flanking region of the SIRT1 gene.

The study protocol was approved by the Ethics Committee of the Faculty of Pharmacy, Cairo University and conformed to the ethical guidelines of the Helsinki Declaration.

### Statistical analysis

Prior to association analyses, deviation from Hardy-Weinberg equilibrium (HWE) was tested for each polymorphism using a Chi-square test. Linkage disequilibrium (LD) calculations were performed using SHEsis software [26], available online http://analysis.bio-x.cn/myAnalysis.php. Data are expressed as means ± S.E. for quantitative variables, and as frequencies and percentages for categorical data. Quantitative variables were compared using independent Student t-test to test for the significance of difference between two means. One way ANOVA was used for analysis of more than two quantitative variables with subsequent multiple comparisons using Tukey–Kramer. To compare categorical data, odds ratios (ORs) of breast cancer associated with each genotype with 95% confidence intervals (CI) were calculated by logistic regression analysis. Logistic regression analyses were also performed to estimate the OR associating different SIRT1 genotypes with clinicopathological characteristics and disease progression. A value of p < 0.05 was considered statistically significant. All statistical calculations were carried out using SPSS (Statistical Package for the Social Sciences) version 20.0 statistical software (SPSS Inc., Chicago, IL, USA).

## Results

The comparison between demographic, laboratory and clinical data of breast cancer patients and control subjects summarized in Table 1 showed no significant difference between normal and breast cancer groups regarding age (p = 0.074), AST (p = 0.310), ALT (p = 0.073), ALP (p = 0.892) and bilirubin (p = 0.785).

**Fig 1:**
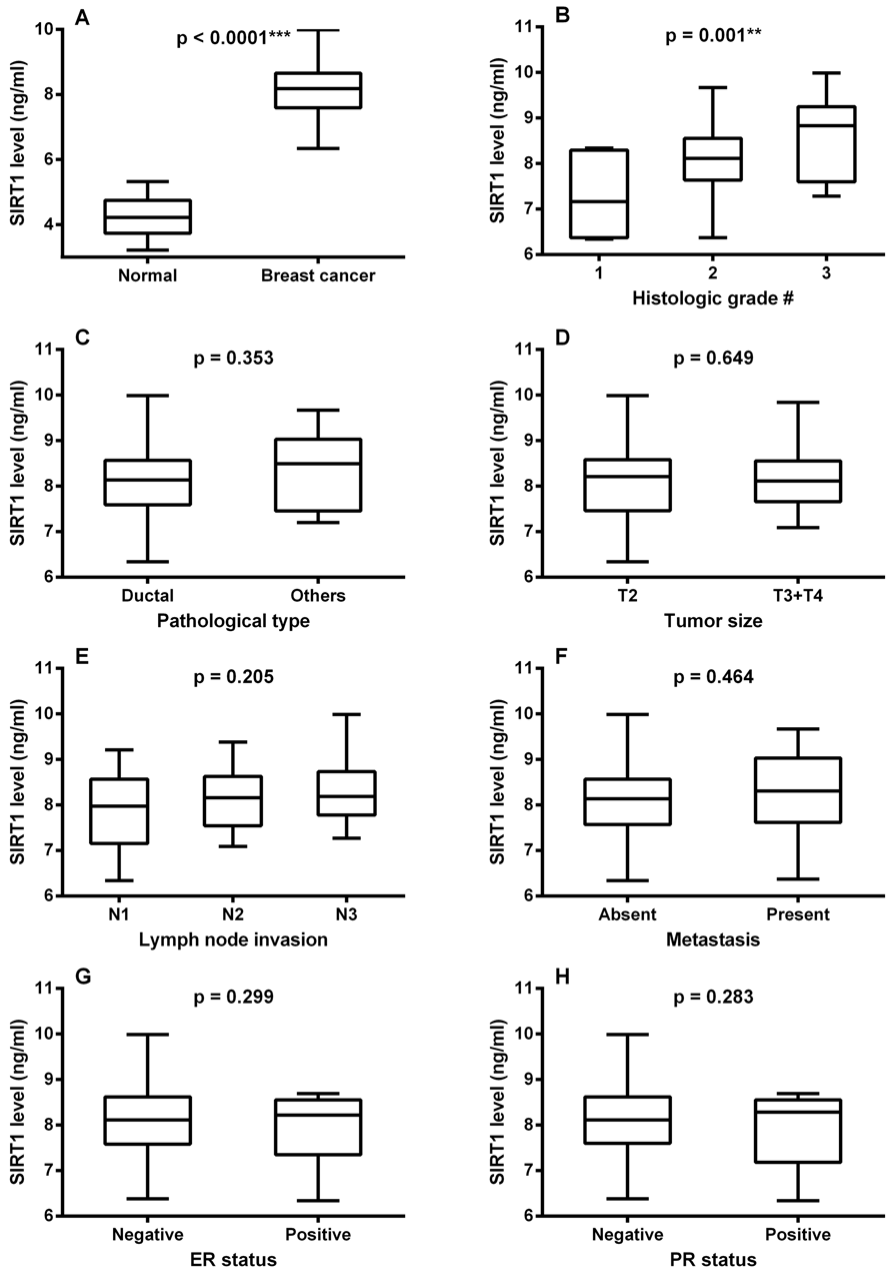
**Box plot of serum SIRT1 levels** (A) in normal subjects and breast cancer patients, (B) with tumor types, (C) with tumor histological grades, (D) with tumor size, (E) with lymph node invasion, (F) in presence and absence of metastasis, (G) with ER status and (H) with PR status. The lines inside the boxes denote the medians. The boxes mark the interval between the 25^th^ and the 75^th^ percentiles. The whiskers denote the interval between the minimum and maximum values. Data are expressed as mean ± S.E. * and *** represent statistical significance at p < 0.05 and p < 0.001, respectively. † modified Bloom–Richardson grading system; T size of the tumor; N regional lymph node involvement; ER, estrogen receptor; PR, progesterone receptor

As shown in Fig 1A, serum SIRT1 level in breast cancer patients was significantly higher than that of normal subjects reaching a value that was almost 2-fold that of the normal control (p < 0.0001). There was also a significant difference between serum SIRT1 levels in different tumor grades, graded according to the modified Bloom–Richardson system [27], as manifested by a higher SIRT1 level in grade 2 compared to grade 1 patients, and a higher value in grade 3 than in grade 2 patients (p < 0.05, Fig 1C). On the other hand, SIRT1 level showed no significant association with other clinicopathological factors. No significant difference was observed between SIRT1 levels in different types of breast cancer (p = 0.353, Fig 1B), neither did they vary significantly with either tumor size (p = 0.649, Fig 1D), lymph node involvement (p = 0.205, Fig 1E), metastasis (p = 0.464, Fig 1F), ER status (p = 0.299, Fig 1G) or PR status (p = 0.283, Fig 1H).

As shown in Table 2, the 3 tested SNPs were in compliance with the Hardy Weinberg equilibrium in control subjects and breast cancer patients (p > 0.05).

**Table 2:** Hardy-Weinberg equilibrium for SIRT1 rs3758391, rs3740051 and rs12778366 in control subjects and breast cancer patients.

		p value	
	rs3758391	rs3740051	rs12778366
**Control subjects**	0.052	0.722	0.076
**Breast cancer patients**	0.170	0.367	0.246

**Fig 2:**
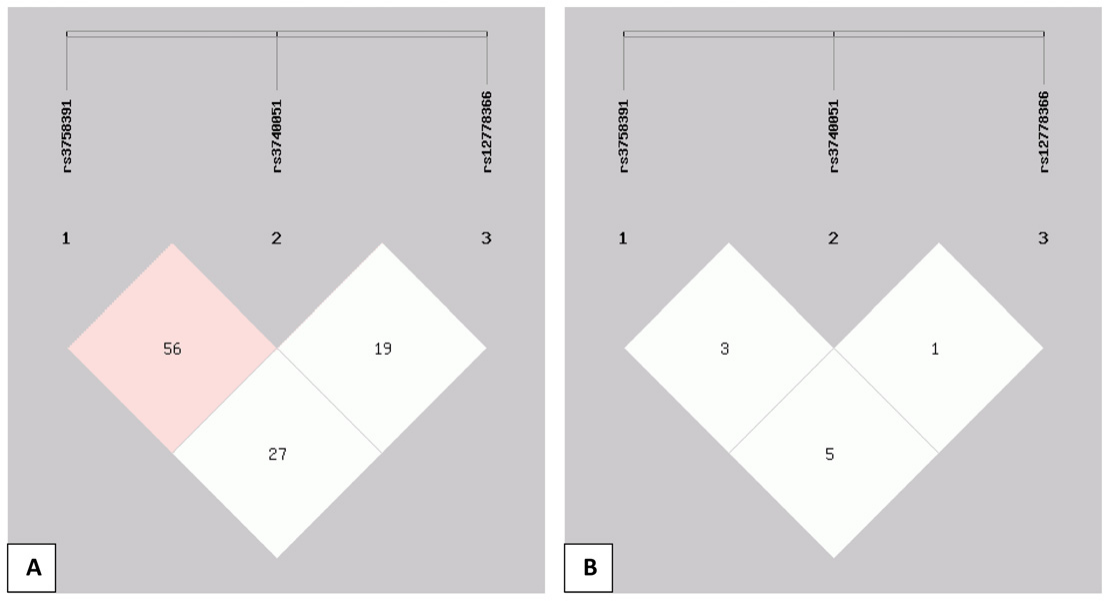
**Linkage disequilibrium structure across the studied SIRT1 single nucleotide polymorphisms (SNPs) rs3758391, rs3740051 and rs12778366:** The pairwise linkage disequilibrium is given for each pair of SNPs showing pairwise D' values (A) and r^2^ values (B) among the 3 SNPs in the present Egyptian population.

Fig 2 shows the linkage disequilibrium structure across the studied SIRT1 SNPs. The pairwise linkage disequilibrium is given for each pair of SNPs showing pairwise D' (Fig 2A) and r^2^ (Fig 2B) values. The observed low r^2^ values indicate that the studied SNPs are not in high linkage disequilibrium despite a D' value of 0.56 for rs3758391 and rs3740051.

Table 3 shows the allele and genotype frequencies of SIRT1 SNPs rs3758391, rs3740051 and rs12778366 in breast cancer patients and control subjects.

**Table 3:** Genotype distribution and allele frequency of SIRT1 variants in control and breast cancer groups. Data are expressed as number and percentage, n (%).

		Control(n = 439)	Breast cancer(n = 541)	OR	95% CI	p value
**rs3758391**						
	***Genotype***					
	TT	149 (33.9)	321 (59.3)	2.840	2.186–3.689	< 0.0001***
	CC+CT	290 (66.1)	220 (40.7)			
	***Allele***					
	C	384 (43.7)	256 (23.7)	2.508	2.067–3.044	< 0.0001***
	T	494 (56.3)	826 (76.3)			
**rs3740051**						
	***Genotype***					
	AA	307 (69.9)	332 (61.4)	1.464	1.120–1.913	0.006**
	GG+AG	132 (30.1)	209 (38.6)			
	***Allele***					
	A	733 (83.5)	843 (77.9)	1.433	1.140–1.802	0.002**
	G	145 (16.5)	239 (22.1)			
**rs12778366**						
	***Genotype***					
	TT	57 (13.0)	305 (56.4)	8.661	6.251–12.001	< 0.0001***
	CC+CT	382 (87.0)	236 (43.6)			
	***Allele***					
	C	538 (61.3)	263 (24.3)	4.928	4.057–5.984	< 0.0001***
	T	340 (38.7)	819 (75.7)			

** and *** represent statistical significance at p < 0.01 and p < 0.001, respectively. OR, odds ratio; CI, confidence intervals

**Fig 3:**
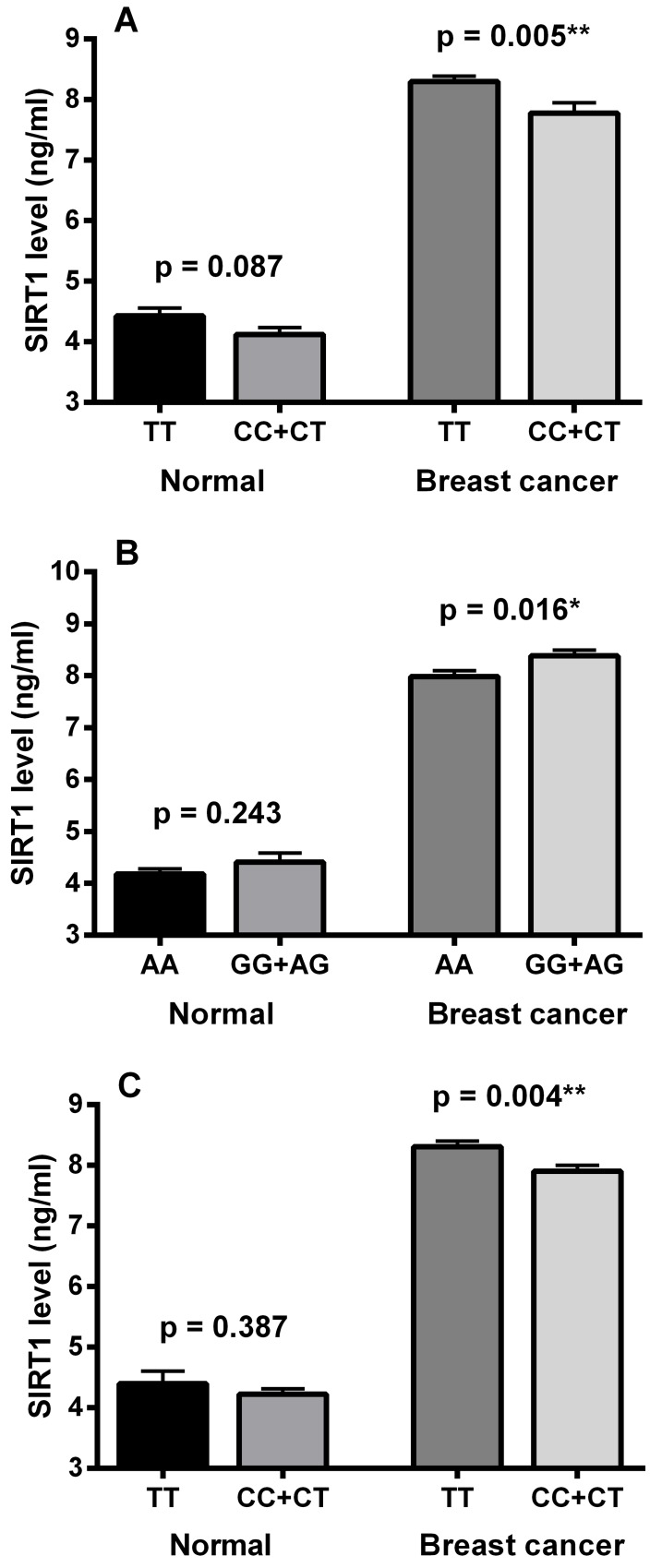
**Serum SIRT1 levels in relation to SIRT1 rs3758391, 3740051 and rs12778366 single nucleotide polymorphisms (SNPs):** (A) SIRT1 levels in TT and combined CC and CT genotypes of rs3758391 SNP, (B) SIRT1 levels in AA and combined GG and AG genotypes of rs3740051 SNP, (C) SIRT1 levels in TT and combined CC and CT genotypes of rs12778366 SNP. Data are expressed as mean ± S.E. * and ** represent statistical significance at p < 0.05 and p < 0.01, respectively.

Concerning SIRT1 SNP rs3758391, a highly significant statistical difference was observed in the T allele frequency and TT genotype distribution between the normal control and breast cancer groups (p < 0.0001). The T allele frequency was markedly higher in breast cancer patients (76%) than in the control group (56%). Also, the homozygous genotype TT was more frequent in the breast cancer group (59%) compared to the control subjects (34%). As indicated in Fig 3A, TT genotype carriers had significantly higher serum SIRT1 levels compared to other rs3758391 genotypes in the breast cancer group (p = 0.005). In normal subjects, serum SIRT1 level was apparently higher in TT genotype carriers than in CC and CT carriers with no statistical significance (p = 0.087).

As regards SIRT1 SNP rs3740051, the data in Table 3 reveal a significant statistical difference in the G allele frequency (p = 0.002) and the combined GG and AG genotype distribution (p = 0.006) between breast cancer and control groups. The G allele was more frequent in breast cancer patients (22%) than in the control group (16.5%). Also, the homozygous genotype GG combined with the heterozygous genotype AG were more frequent in the breast cancer group (39%) than in control subjects (30%). Additionally, as shown in Fig 3B, the combined GG and AG genotypes exhibited significantly higher serum SIRT1 levels than the AA genotype in the breast cancer group (p = 0.016). On the other hand, no significant difference was observed in serum SIRT1 levels between the corresponding genotypes in the control group (p = 0.243).

Regarding the SIRT1 locus rs12778366, Table 3 shows a highly significant statistical difference in the T allele frequency and TT genotype distribution between the two groups (p < 0.0001). The T allele frequency was markedly higher in breast cancer patients than in the control group (76% versus 39%). Also, the homozygous genotype TT was considerably more frequent in the breast cancer group compared to the control group (56% versus 13%). In addition, TT genotype carriers showed significantly higher serum SIRT1 levels when compared with CC and CT carriers in the breast cancer group (p = 0.004), whereas no significant difference was found between the corresponding genotypes in the control group (p = 0.387) as illustrated in Fig 3C.

In Tables 4–6, the distributions of the rs3758391, rs3740051 and rs12778366 genotypes were compared in relation to the different clinicopathological variables associated with breast cancer.

**Table 4:** Association of SIRT1 rs3758391 gene polymorphism with breast cancer clinicopathological variables. Data are expressed as number and percentage, n (%).

Characteristics		TT	CC+CT	OR	95% CI	p value
**Type**						
	**Ductal**	282 (60.1)	187 (39.9)	1.276	0.775–2.102	0.368
	**Others**	39 (54.2)	33 (45.8)			
**Histological grade**†						
	**1**	5 (14.3)	30 (85.7)	7.709	2.932–20.275	< 0.0001*** ^***a***^
	**2**	230 (56.2)	179 (43.8)	6.085	3.153–11.743	< 0.0001*** ^***b***^
	**3**	86 (88.7)	11 (11.3)	46.909	15.060–146.110	< 0.0001*** ^***c***^
**Tumor size**						
	**T2**	201 (57.9)	146 (42.1)	1.178	0.822–1.688	0.412
	**T3+T4**	120 (61.9)	74 (38.1)			
**Lymph node invasion**						
	**N1**	30 (27.8)	78 (72.2)	4.705	2.900–7.633	< 0.0001*** ^***a***^
	**N2**	190 (64.4)	105 (35.6)	1.509	0.966–2.356	0.079 ^***b***^
	**N3**	101 (73.2)	37 (26.8)	7.097	4.034–12.488	< 0.0001*** ^***c***^
**Metastasis**						
	**Absence**	245 (58.1)	177 (41.9)	1.277	0.838–1.946	0.291
	**Presence**	76 (63.9)	43 (36.1)			
**ER status**						
	**Negative**	257 (57.9)	187 (42.1)	1.411	0.890–2.236	0.171
	**Positive**	64 (66.0)	33 (34.0)			
**PR status**						
	**Negative**	262 (58.0)	190 (42.0)	1.426	0.885–2.299	0.158
	**Positive**	59 (66.3)	30 (33.7)			

*** represents significant difference at p < 0.001.

^***a***^ 2 versus 1

^***b***^ 3 versus 2

^***c***^ 3 versus 1

† modified Bloom–Richardson grading system; T size of the tumor; N regional lymph node involvement; ER, estrogen receptor; PR, progesterone receptor

**Table 5:** Association of SIRT1 rs3740051 gene polymorphism with breast cancer clinicopathological variables. Data are expressed as number and percentage, n (%).

Characteristics		AA	GG+AG	OR	95% CI	p value
**Type**						
	**Ductal**	285 (60.8)	184 (39.2)	1.214	0.722- 2.041	0.517
	**Others**	47 (65.3)	25 (34.7)			
**Histological grade**†						
	**1**	19 (54.3)	16 (45.7)	1.485	0.741–2.976	0.277 ^***a***^
	**2**	261 (63.8)	148 (36.2)	0.655	0.419–1.025	0.081 ^***b***^
	**3**	52 (53.6)	45 (46.4)	0.973	0.448–2.114	1.000 ^***c***^
**Tumor size**						
	**T2**	204 (58.8)	143 (41.2)	0.736	0.510–1.061	0.118
	**T3+T4**	128 (66.0)	66 (34.0)			
**Lymph node invasion**						
	**N1**	66 (61.1)	42 (38.9)	0.955	0.608–1.500	0.909 ^***a***^
	**N2**	177 (60.0)	118 (40.0)	1.211	0.796–1.842	0.398 ^***b***^
	**N3**	89 (64.5)	49 (35.5)	1.156	0.687–1.946	0.597 ^***c***^
**Metastasis**						
	**Absence**	267 (63.3)	155 (36.7)	1.431	0.948–2.160	0.089
	**Presence**	65 (54.6)	54 (45.4)			
**ER status**						
	**Negative**	289 (65.1)	155 (34.9)	2.341	1.499–3.656	0.0002***
	**Positive**	43 (44.3)	54 (55.7)			
**PR status**						
	**Negative**	299 (66.2)	153 (33.8)	3.316	2.068–5.318	< 0.0001***
	**Positive**	33 (37.1)	56 (62.9)			

*** represents significant difference at p < 0.001.

^***a***^ 2 versus 1

^***b***^ 3 versus 2

^***c***^ 3 versus 1

† modified Bloom–Richardson grading system; T size of the tumor; N regional lymph node involvement; ER, estrogen receptor; PR, progesterone receptor

**Table 6:** Association of SIRT1 rs12778366 gene polymorphism with breast cancer clinicopathological variables. Data are expressed as number and percentage, n (%).

Characteristics		TT	CC+CT	OR	95% CI	p value
**Type**						
	**Ductal**	265 (56.5)	204 (43.5)	1.039	0.631–1.713	0.899
	**Others**	40 (55.6)	32 (44.4)			
**Histological grade**†						
	**1**	5 (14.3)	30 (85.7)	8.435	3.207–22.190	< 0.0001 *** ^***a***^
	**2**	239 (58.4)	170 (41.6)	1.205	0.764–1.903	0.491 ^***b***^
	**3**	61 (62.9)	36 (37.1)	10.167	3.620–28.556	< 0.0001 *** ^***c***^
**Tumor size**						
	**T2**	190 (54.8)	157 (45.2)	0.831	0.582–1.187	0.321
	**T3+T4**	115 (59.3)	79 (40.7)			
**Lymph node invasion**						
	**N1**	44 (40.7)	64 (59.3)	2.151	1.373–3.370	0.001** ^***a***^
	**N2**	176 (59.7)	119 (40.3)	1.084	0.716–1.641	0.752 ^***b***^
	**N3**	85 (61.6)	53 (38.4)	2.333	1.394–3.904	0.001** ^***c***^
**Metastasis**						
	**Absence**	229 (54.3)	193 (45.7)	0.671	0.441–1.022	0.075
	**Presence**	76 (63.9)	43 (36.1)			
**ER status**						
	**Negative**	263 (59.2)	181 (40.8)	1.903	1.220–2.967	0.005**
	**Positive**	42 (43.3)	55 (56.7)			
**PR status**						
	**Negative**	268 (59.3)	184 (40.7)	2.047	1.290–3.248	0.002**
	**Positive**	37 (41.6)	52 (58.4)			

** and *** represent significant difference at p < 0.01 and p < 0.001, respectively.

^***a***^ 2 versus 1

^***b***^ 3 versus 2

^***c***^ 3 versus 1

† modified Bloom–Richardson grading system; T size of the tumor; N regional lymph node involvement; ER, estrogen receptor; PR, progesterone receptor

As depicted in Table 4, statistical analysis of the correlation between SIRT1 rs3758391 and clinicopathological parameters in breast cancer patients revealed highly significant associations with histologic grade and lymph node status. The distribution of the TT genotype increased significantly (p < 0.0001) with increasing the tumor grade (14% in grade 1, 56% in grade 2 and 89% in grade 3). Also, the TT genotype was significantly more frequent in N2 and N3 patients than in N1 patients (64% in N2 patients and 73% in N3 patients versus 28% in N1 patients, p < 0.0001).

Table 5 shows the SIRT1 rs3740051 genotype distributions in relation to different clinicopathological variables in breast cancer. SIRT1 rs3740051 polymorphism was significantly associated with both ER (p = 0.0002) and PR expression (p < 0.0001) as manifested by a higher distribution of combined GG and AG genotypes in ER positive than in ER negative patients (56% versus 35%) and in PR positive than in PR negative patients (63% versus 34%). On the other hand, the AA genotype was notably associated with negative ER and PR status whereby AA carriers represented 65% of ER negative patients and 66% of PR negative patients versus 44% of ER positive patients and 37% of PR positive patients.

SIRT1 rs12778366 genotype frequencies with respect to different clinicopathological features are represented in Table 6. The TT genotype was significantly associated with histologic grade, lymph node, ER and PR states. Such correlations were evidenced by higher frequencies in grades 2 and 3 than in grade 1 (58% for grade 2 and 63% for grade 3 versus 14% for grade 1, p < 0.0001), in N2 and N3 than in N1 states (60% for N2 and 62% for N3 versus 41% for N1, p = 0.001), in ER negative than in ER positive patients (59% versus 43%, p = 0.005) and in PR negative than in PR positive patients (59% versus 42%, p = 0.002).

## Discussion

To our knowledge, previous studies have explored the association of SIRT1 expression with prognosis in breast cancer [28–30], but they did not consider the role of SIRT1 gene polymorphism in breast cancer risk or prognosis.

This is the first study to assess the association between genetic variation in SIRT1 and susceptibility and prognosis of breast cancer. The genotype distribution and allele frequency of SIRT1 promoter region polymorphisms rs3758391, rs3740051 and rs12778366 were compared between breast cancer patients and normal subjects. Also, the association between the studied SNPs and clinicopathological features of breast cancer was investigated. Our findings suggest a role of genetic variation in SIRT1 at the studied loci as a risk factor and as a prognostic factor for breast cancer in the Egyptian population.

The present investigation revealed considerably higher serum SIRT1 levels in breast cancer patients than in normal subjects. Up-regulation of SIRT1 has been reported in various human malignancies including breast cancer, prostate cancer, lymphoma, colon cancer, gastric cancer and hepatocellular carcinoma [9,28,29,31–34]. Our findings of elevated SIRT1 levels in breast cancer patients are in agreement with those of Sung et al. [8] who demonstrated higher SIRT1 expression in tumor breast tissues than in matched normal tissues at the protein level. On the contrary, Cao et al. showed significantly lower expression level of SIRT1 protein in breast cancer than in normal tissues [35]. In addition, Wang et al. [13] reported a decrease in SIRT1 expression in breast tumor tissue.

Although the role of SIRT1 in breast cancer and its association with outcome have been debated because of conflicting reports of its activity, the present findings of substantially higher serum SIRT1 levels in breast cancer patients compared to normal control subjects suggest a tumor promoting role for SIRT1 in breast cancer in the studied patients. The oncogenic role of SIRT1 is further supported by our observation of significant association of breast cancer with the genotypes having higher SIRT1 levels in each of the studied SNPs. In agreement with our suggestion, many recent reports have shown that SIRT1 has a tumor promoting role [36–39]. Up-regulation of SIRT1 induces deacetylated inactivation of p53, which allows cells to proliferate in the presence of damaged DNA such that mutations accumulate, including those in p53 itself, which disrupt cell cycle control and promote tumor progression [39,38,37]. As pointed out by Roth and Chen [15], despite the controversy over the role of SIRT1 in tumorigenesis, it appears that SIRT1 has a consistent role in mediating cancer cell survival.

Comparing SIRT1 rs3758391 genotype and allele distributions between breast cancer patients and normal subjects revealed significantly higher frequencies of the TT genotype and the T allele in breast cancer patients than in normal controls. These findings indicate the association of SIRT1 gene polymorphism rs3758391 with breast cancer and signify the SIRT1 rs3758391 T allele as being a potential risk factor for breast cancer. Furthermore, serum SIRT1 level in TT genotype carriers was significantly higher than in other genotype carriers in the breast cancer group. A significant association was previously reported between the rs3758391 TT genotype and a higher mRNA expression of SIRT1 in healthy individuals [40], an observation which was only apparent in our normal controls but highly significant in breast cancer patients regarding serum SIRT1 level. Consiglio et al. suggested that, in systemic lupus erythematosus, the SIRT1 rs3758391 T allele differentially induces SIRT1 expression, influencing histone acetylation levels in CD4+ lymphocytes [41]. However, the correlation between the T allele and a higher SIRT1 expression is controversial, since Rai et al. were unable to find any difference in SIRT1 expression in relation to rs3758391 genotypes in a North Indian population [19].

Regarding the rs3740051 SNP, the combined GG and AG genotypes were significantly more frequent in breast cancer patients than in control subjects. Interestingly, both genotypes exhibited, together, a significantly higher serum SIRT1 level compared with the AA genotype in the breast cancer group. In addition, the frequency of the G allele was significantly higher in the breast cancer group than in normal subjects. These data implicate SIRT1 rs3740051 as a possible contributor to breast tumorigenesis, and suggest a role for the G allele in the increased expression of SIRT1 and higher susceptibility to breast cancer.

In the current investigation, a highly significant association was observed between SIRT1 rs12778366 and breast cancer as evidenced by significantly higher TT genotype distribution and T allele frequency in breast cancer patients compared to normal control subjects. These results imply the potential involvement of the T allele of SIRT1 rs12778366 SNP as a risk factor for breast cancer. These findings were paralleled by the observation of significantly higher serum SIRT1 levels in the TT genotype carriers than in CC and CT genotype carriers in breast cancer patients but not in normal subjects. Elevated SIRT1 levels observed in the TT genotype of the rs12778366 SNP in the breast cancer group are in harmony with the results of Rai et al. who demonstrated that the high-expressing TT genotype of the promoter region polymorphism rs12778366 may affect the expression profile of SIRT1 [19]. At the same time, lack of significant difference in SIRT1 levels among different genotypes of healthy subjects is consistent with the report of Hu et al. who stated that the rs12778366 genotypes were not associated with SIRT1 mRNA expression in healthy individuals [40].

Altogether, it could be inferred that the marked elevation in circulating SIRT1 levels in breast cancer patients might be attributed to the significant presence of the high risk and high SIRT1-expressing rs3758391 and rs12778366 TT genotypes as well as the rs3740051 G allele carriers in breast cancer patients as compared to normal control subjects. On the other hand, in the normal group, there seems to be a tendency of SIRT1 level elevation particularly in rs3758391 TT genotype carriers, compared to CC and CT carriers (p = 0.087), and to a lesser extent in rs12778366 TT carriers, compared to CC and CT carriers, and in rs3740051 combined GG+AG carriers, compared to AA carriers. However, such minor elevations failed to have a significant impact on serum SIRT1 levels in the whole set of normal subjects and, hence, could not reach statistical significance due to the markedly low frequencies of the high-expressing genotypes in the normal group.

Regarding rs3758391 and rs3740051 SNPs, the allele frequencies observed in our control group were comparable to those in other populations (Gujarati Indians in Houston, Texas and Mexican ancestry in Los Angeles, California), based on data from the HapMap project, version 2010–08_phaseII+III. On the other hand, the rs12778366 allele frequencies in healthy subjects in our study were quite different from those reported in other populations. Egyptian control subjects in the present study showed substantially higher C allele frequencies and markedly lower T allele frequencies than typically observed in any other population reported to date. Moreover, the T allele frequency in breast cancer patients was relatively close to that reported in normal subjects of other populations. Inconsistent patterns of allele frequencies among Egyptians in comparison with other populations have been previously reported by **Shahin et al. (2011)** [42] who observed variant alleles in Egyptians at CYP2C9 *4, *5, and *8, along with CALU rs339097, all of which are generally considered nonvariant in Caucasians, and observed primarily in those of African ancestry. In contrast, the minor allele frequencies for CYP2C9 *2 and *3 were similar to Caucasians. CYP4F2 V433M was substantially higher than any other population reported to date, whereas APOE rs429358 was lower than typically observed in African–Americans or Caucasians. The authors attributed such inconsistencies to the extensive genetic admixture of the Egyptian population. In addition to the fact that the African populations in general are the most genetically diverse since humans originated in Africa and spread into Europe and then the Asian and American continents [43], the Egyptian population, in particular, is one that has undergone extensive genetic admixture and racial mixing, which created a heavily mixed population of modern Egyptians [44]. Altogether, our findings reinforce the conclusion of **Shahin et al. (2011)** [42] that polymorphism frequencies in Egyptians cannot be presumed to mirror those of any other continental population. We are not aware of any population genetics studies that clearly define the ancestral makeup of Egyptians, and so the findings in this study further support recent recommendations of the importance of genomics research in the Mediterranean region [45].

Our observations regarding serum SIRT1 levels provide a potential mechanism for the association of the studied SNPs with genetic susceptibility to breast cancer. It is possible to predict that the observed elevation in serum SIRT1 level in breast cancer patients could be attributed to the up-regulation of SIRT1 gene expression. Similarly, Kilic and co-workers (2014), who were the first to report an association between SIRT1 gene polymorphisms and the plasma levels of SIRT1, attributed the observed alterations in plasma SIRT1 levels to the modulation of SIRT1 gene expression by SIRT1 SNPs [46]. Our suggestion that up-regulated SIRT1 gene expression is the cause of elevated serum SIRT1 levels observed in our study is based on several factors. Firstly, SIRT1 up-regulation has been previously demonstrated in breast cancer [8,28]. Secondly, there are several reports on the roles of SIRT1 promoter region polymorphisms rs3758391, rs3740051 and rs12778366 in the differential expression of SIRT1 [18–21,40,41,47]. Thirdly and most importantly, our observations of significantly higher serum levels of SIRT1 in breast cancer patients reveal a genotype-dependent pattern. Given that SIRT1 rs3758391 TT, rs12778366 TT and rs3740051 combined GG and AG genotypes were significantly associated with breast cancer and showed higher SIRT1 levels compared to other genotypes, we could speculate that the association of SIRT1 rs3758391 T allele, rs12778366 T allele and, to a lesser extent, rs3740051 G allele with a higher breast cancer risk may be explained by higher levels of SIRT1 expression, possibly having an impact on histone deacetylation. The presently observed contribution of the rs3758391 and rs12778366 TT genotypes to higher serum SIRT1 levels is in accordance with the reported association of these genotypes with high SIRT1 expression [19,40,41]. Nevertheless, the effects of other factors on serum SIRT1 level, such as excessive cell damage and rupture, rather than up-regulated gene expression, could not be ruled out. Therefore, it would be of great value to study—in future investigations—the effect of the studied SNPs on SIRT1 gene and protein expression levels in breast cancer tissue.

In the present study, both SIRT1 SNPs rs3758391 and rs12778366 were associated with histologic grade and lymph node invasion in breast cancer patients, as manifested by significantly higher frequencies of the TT genotype with higher tumor grades and higher lymph node involvement. Such findings highlight the potential prognostic values of SIRT1 rs3758391 and rs12778366 gene polymorphisms in breast cancer.

The presently noticed association of rs3758391 and rs12778366 TT genotypes with histologic grade could be attributed, at least partly, to their significantly higher SIRT1 levels compared to CC and CT genotypes. Such suggestion is based on the reported positive relation between SIRT1 expression and tumor proliferation [30]. Additionally, SIRT1 expression was significantly associated with poorer differentiation status in advanced non-small cell lung cancer, indicating that SIRT1 expression was parallel with the tumor severity [48]. Correlation of tumor grade with SIRT1 level was evident in the present study by significantly higher SIRT1 levels in grade 2 than in grade 1 and in grade 3 than in grade 2. In contrast, Sung et al. reported that up-regulation of SIRT1 in breast tumor tissue was significantly associated with low grade breast cancer [8].

The significantly higher SIRT1 levels noted in the TT genotypes of the SNPs rs3758391 and rs12778366, compared to the CC and CT genotypes, could also account for the presently detected association between the TT genotypes and lymph node status. Such suggestion is based on the previously reported correlation between SIRT1 expression and lymph node status in breast cancer [30,35]. Although the TT genotypes, showing elevated SIRT1 levels, correlated with lymph node status, we failed to show a direct association between serum SIRT1 levels and lymph node status. Differences in SIRT1 levels were significant only among the genotypes of each SNP separately rather than among the whole set of patients in terms of their lymph node status.

Explaining the correlations of SIRT1 rs3758391 and rs12778366 TT genotypes with histologic grade and lymph node status on the basis of their elevated SIRT1 protein levels is in accordance with the report of Cao et al. that SIRT1 expression is strongly correlated with aggressive tumor behavior [49]. Actually, several studies have indicated that overexpression of SIRT1 may correlate with poor prognosis in breast carcinoma [28–30].

Although our results failed to show a significant difference in SIRT1 levels with different ER or PR status, SIRT1 rs3740051 and rs12778366 gene polymorphisms showed significantly different genotype distributions with ER and PR expression. The currently observed associations of rs3740051 and rs12778366 polymorphisms with ER and PR status might be linked to the significant variation in SIRT1 levels among different genotypes of each SNP. Actually, previous reports linked ER and PR status to expression of SIRT1 in breast cancer tissue [8,30,35].

Concerning the rs3740051 SNP, the AG and GG genotypes together were significantly associated with positive ER and PR status. Association of ER and PR expression with the AG and GG genotypes, showing high SIRT1 levels, appears to be in agreement with a report by Sung et al. indicating a significant correlation of ER and PR expression with the up-regulation of SIRT1 in breast tumor tissue [8]. Conversely, the AA genotype was significantly more frequent in ER and PR negative than in ER and PR positive patients. Our findings of a significant association of the AA genotype with negative PR status together with the lower SIRT1 level observed in such genotype, compared with the AG and GG genotypes, seem to be in accordance with the results of Cao et al. who showed that low SIRT1 expression in breast cancer tissue was markedly associated with negative PR status [35].

The SIRT1 rs12778366 SNP significantly correlated with both PR and ER statuses. Surprisingly, the TT genotype, showing markedly elevated SIRT1 level compared to CC and CT genotypes, was significantly associated with negative ER and PR status. Such observations seem to contradict the results of Sung et el. who demonstrated that ER and PR expression significantly correlated with the overexpression of SIRT1 in breast cancer tissue [8].

Considering that breast cancer is a complex disease triggered by myriad genetic and lifestyle factors, the underlying mechanisms are extremely complicated and remain largely unknown. However, the controversial findings might be the result of different patient populations, numbers of samples and/or ethnic variations. The conflicting observations might also emerge from consideration of the complex network of proteins in which SIRT1 is a hub [50,51]. Furthermore, the effect of SIRT1 may vary according to the accompanying mutation status of SIRT1 gene or other tumor-related genes [48] or due to differences in downstream targets of the enzyme [28]. Therefore, further research is needed to clarify the function of SIRT1 and more samples are required to resolve such inconsistencies.

## Conclusions

Our study is the first to report an association of SIRT1 gene polymorphism, namely rs3758391 and rs12778366, and, to a lesser extent rs3740051, with breast cancer risk, with the T allele (for rs3758391 and rs12778366) and the G allele (for rs3740051) being potential risk factors. Our study also highlights a potential role for rs3758391 and rs12778366 SNPs in breast cancer prognosis. Accordingly, the current findings would highly recommend that women, particularly Egyptians, with a strong family history of breast cancer should be genetically tested for SIRT1 rs3758391 and rs12778366 genetic variants to predict risk of breast cancer. Further functional studies are necessary to investigate the possible mechanisms underlying these potential associations. Our findings of elevated serum SIRT1 levels in breast cancer patients, being more pronounced in high risk genotypes that are reportedly high SIRT1-expressing genotypes, have important implications for future studies to elucidate the mechanism of regulation of SIRT1 levels by the studied SIRT1 promoter region polymorphisms. SIRT1 expression and its role in breast tumorigenesis could be influenced by other functional variants in the gene. Hence, a thorough exploration of SIRT1 gene variations in the Egyptian population and extension of the present work in a larger data set is warranted for confirming our preliminary conclusion and recommendation since a major limitation to our study is the relatively small sample size. It would also be tempting to investigate whether the current observations would replicate in other population groups.

## Supporting Information

S1 Table Genotypes. (XLSX)
